# Public Health Impact of Complete and Incomplete Rotavirus Vaccination among Commercially and Medicaid Insured Children in the United States

**DOI:** 10.1371/journal.pone.0145977

**Published:** 2016-01-11

**Authors:** Girishanthy Krishnarajah, Mei Sheng Duh, Caroline Korves, Kitaw Demissie

**Affiliations:** 1 GlaxoSmithKline Vaccines, King of Prussia, Pennsylvania, United States of America; 2 Analysis Group, Inc., Boston, Massachusetts, United States of America; 3 Rutgers University, School of Public Health, Piscataway, New Jersey, United States of America; 4 Rutgers Robert Wood Johnson Medical School, New Brunswick, New Jersey, United States of America; Groningen Research Institute of Pharmacy, NETHERLANDS

## Abstract

**Background:**

This study (**NCT01682005**) aims to assess clinical and cost impacts of complete and incomplete rotavirus (RV) vaccination.

**Methods:**

Beneficiaries who continuously received medical and pharmacy benefits since birth were identified separately in Truven Commercial Claims and Encounters (2000–2011) and Truven Medicaid Claims (2002–2010) and observed until the first of end of insurance eligibility or five years. Infants with ≥1 RV vaccine within the vaccination window (6 weeks-8 months) were divided into completely and incompletely vaccinated cohorts. Historically unvaccinated (before 2007) and contemporarily unvaccinated (2007 and after) cohorts included children without RV vaccine. Claims with International Classification of Disease 9^th^ edition (ICD-9) codes for diarrhea and RV were identified. First RV episode incidence, RV-related and diarrhea-related healthcare resource utilization after 8 months old were calculated and compared across groups. Poisson regressions were used to generate incidence rates with 95% confidence intervals (CIs). Mean total, inpatient, outpatient and emergency room costs for first RV and diarrhea episodes were calculated; bootstrapping was used to construct 95% CIs to evaluate cost differences.

**Results:**

1,069,485 Commercial and 515,557 Medicaid patients met inclusion criteria. Among commercially insured, RV incidence per 10,000 person-years was 3.3 (95% CI 2.8–3.9) for completely, 4.0 (95% CI 3.3–5.0) for incompletely vaccinated, and 20.9 (95% CI 19.5–22.4) for contemporarily and 40.3 (95% CI 38.6–42.1) for historically unvaccinated. Rates in Medicaid were 7.5 (95% CI 4.8–11.8) for completely, 9.0 (95% CI 6.5–12.3) for incompletely vaccinated, and 14.6 (95% CI 12.8–16.7) for contemporarily and 52.0 (95% CI 50.2–53.8) for historically unvaccinated. Mean cost for first RV episode per cohort member was $15.33 (95% CI $12.99-$18.03) and $4.26 ($95% CI $2.34-$6.35) lower for completely vaccinated versus contemporarily unvaccinated in Commercial and Medicaid, respectively.

**Conclusions:**

RV vaccination results in significant reduction in RV infection. There is evidence of indirect benefit to unvaccinated individuals.

## Background

Rotavirus (RV) is the most common cause of severe diarrhea among children less than five years of age worldwide, accounting for 453,000 deaths each year [1]. Although rarely fatal in the United States (US), RV remains a major cause of severe dehydration and hospitalization in children, thus incurring substantial healthcare resource utilization and costs. Before the initiation of a routine infant RV vaccination program in February 2006, nearly every child in the US was infected with RV by five years of age, resulting annually in approximately 410,000 outpatient visits (doctors’ office visits), 205,000–272,000 emergency room (ER) visits, 55,000–70,000 hospitalizations, and total annual direct and indirect costs of approximately $1 billion [2–7].

Both the Advisory Committee on Immunization Practices (ACIP) and the American Academy of Pediatrics recommend routine immunization of infants with RV vaccine [7,8]. Two brands of oral RV vaccines are currently approved by the US Food and Drug Administration (FDA): *Rotateq*^®^ (registered trademark of Merck MSD, approved in 2006 in US) and *Rotarix*^®^ (registered trademark of the GlaxoSmithKline group of companies, approved in 2008 in US). The vaccine is administered in two doses if *Rotarix*^®^ is used; and in three doses if either *Rotateq*^®^ or a mix of brands (i.e., both *Rotarix*^®^ and *Rotateq*^®^) is used. Irrespective of the brand of vaccine used, the ACIP recommends a vaccination window from six weeks old to eight months and zero days old [7]. The National Immunization Survey (NIS) among children 19–35 months of age indicated that 67.3% received either at least two doses of *Rotarix*^®^ or at least three doses of *Rotateq*^®^ in 2011, and 68.6% did so in 2012 [9].

Estimates of the clinical and economic impact of RV vaccination (including both *Rotateq*^®^ and *Rotarix*^®^) among children aged less than five years, particularly during recent years’ RV seasons, are limited. In addition, published studies to date using real-world practice data to evaluate complete and partial vaccine effectiveness have included only the *Rotateq*^®^ vaccine, and they have not analyzed the Medicaid low-income population [10–13]. Medicaid is the main public health insurance program for the low-income population in the US, covering 16% of the population for the years 2011–2012; 53% of the population was insured through employer sponsored insurance or other private insurance during this time [14]. In June 2013, more than 28 million children were enrolled in Medicaid and another 5.7 million enrolled in the Children’s Health Insurance Program (CHIP) which provides coverage to children in families with incomes too high to qualify for Medicaid but unable to afford private insurance [15]. Medicaid and other public programs provide insurance to a significant proportion of children in the US, though the enrollment varies considerably by race: more than half of Hispanic and non-Hispanic Black children in the US are enrolled in Medicaid or another public program, and approximately one quarter of Asian and non-Hispanic White children in the US are enrolled in these programs [16].

The current study looks at the use of currently available RV vaccines in actual clinical practice in both the privately insured and Medicaid insured populations. Given the differences in the privately and Medicaid insured populations in terms of income and race and the inability to adjust for these factors, the current study examined outcomes separately in each population.

The objectives of this study were to describe and compare clinical and economic outcomes among completely vaccinated, incompletely vaccinated, and unvaccinated children following the ACIP recommended vaccination window. Outcomes were also compared between unvaccinated children under five years after the availability of vaccination to a similarly aged cohort of unvaccinated children prior to the availability of vaccination to ascertain the indirect benefits of the vaccine. As the ACIP recommended vaccination window concludes at 8 months and zero days, follow-up to assess RV clinical and economic outcomes by vaccination status began at 8 months. As a sensitivity analysis, RV clinical and economic outcomes by vaccination status were also assessed during the vaccination window, from 6 weeks to 8 months of age.

## Methods

### Data Source

Data from the 2000–2011 Truven Commercial Claims and Encounters and the 2002–2010 Truven Medicaid databases were analyzed separately. Truven data are derived from insurance claims and contain de-identified information from various public and private health plans, including: comprehensive, exclusive provider organizations, health maintenance organizations, non-capitated point-of-service, preferred provider organizations, capitated or partially-capitated point-of-service, consumer-driven health plans, and high deductible health plans.

The Truven Commercial database contains claims from enrollees of approximately 100 employers and a number of health plans. During the study period of 2000–2011, data from approximately nine million children under five years of age were captured. The Truven Medicaid database contains the pooled healthcare experience of approximately 7 million Medicaid enrollees from seven state contributors and five Medicaid health plans. During the study period of 2002–2010, data from approximately 4.5 million children under five years of age were captured. In both databases, information is available on patient demographics, date of service, place of service (hospital, ER, urgent care facility, outpatient clinic), International Classification of Diseases 9^th^ edition (ICD-9) codes, Current Procedural Terminology (CPT) codes, length of hospital stay, and cost data in the form of payments made by insurers and patients (copay) to providers (i.e., physicians and hospitals). The data included in the Truven database are de-identified and are in compliance with the Health Insurance Portability and Accountability Act of 1996 to preserve participant anonymity and confidentiality. The study was reviewed and approved by the Institutional Review Board of the University of Medicine and Dentistry of New Jersey.

### Study Population

To be included in the study population, beneficiaries must have continuously received both medical and pharmacy benefits (to ensure complete claims data) from birth to age 8 months (or beyond) to assess RV vaccination during the ACIP recommended vaccination window. Due to the absence of birth dates in third party data cuts in Truven insurance claims data, the enrolment date was used as a proxy for birth date when year of first enrolment and birth year were equivalent. Beneficiaries who enrolled in capitation-based health plans, and therefore may have incomplete claims, were excluded. Individuals with RV-coded encounters identified by claims with the specific ICD-9 code for RV (008.61 -Enteritis due to rotavirus) prior to 8 months of age were excluded from the analysis as incidence of first episodes of RV following the vaccination window was a main outcome of interest.

### Study Design

A retrospective, longitudinal, open-cohort design was used for this study. Each eligible child was observed from his/her estimated birth date to end of continuous eligibility (i.e., due to disenrollment, data cut-off, or death) or five years of age, whichever occurred first. Individuals’ person-time was partitioned into the periods from 6 weeks through 8 months old to assess exposure to RV vaccines during the vaccination window, and from 8 months old and onwards to assess clinical and economic outcomes. Each child has a unique identifier in the database allowing for capture of longitudinal data.

### Vaccination Cohorts

Children who received two *Rotarix*^®^, three *Rotateq*^*®*^ or three mixed type vaccinations during the vaccination window contributed person-time to the complete vaccination cohort; children who received less than complete vaccination but at least one during the vaccination window contributed person-time to the incomplete vaccination cohort. The historically unvaccinated cohort was for individuals who were 8 months old prior to 2007 (and thus reached the end of the vaccination window either in the year that RV vaccine was introduced or else prior) and received no vaccination during the window. The contemporarily unvaccinated cohort was for individuals 8 months old after 2006 with no vaccination during the window. For the period from 8 months old and onwards, claims with the ICD-9 codes for diarrhea and/or RV were identified and used to describe the clinical and economic burden of RV by vaccination status. Comparisons between contemporarily and historically unvaccinated children who were similarly aged were done to assess indirect effect of vaccination which occurs when unvaccinated individuals gain protection against infection when others are vaccinated and thus less likely to acquire and transmit infection.

### Study Outcomes

#### Clinical Outcomes

Clinical outcomes of interest included first episode of RV infection, RV-coded healthcare encounters (i.e., hospitalizations, outpatient visits, and ER visits), and diarrhea-coded healthcare encounters. As testing for RV is not performed routinely on all patients with diarrhea, claims coded as RV likely represent only a fraction of all RV-related encounters [10,17]; thus diarrhea-coded healthcare encounters were also of interest. RV-coded encounters were identified by claims with the specific ICD-9 code for RV (008.61, enteritis due to rotavirus). The first occurrence of an RV-coded encounter marked incidence of first RV episode. Diarrhea-coded encounters were identified by claims with the non-specific ICD-9 codes for diarrhea: 009.0–009.3 (presumed infectious gastroenteritis), 558.9 (presumed noninfectious gastroenteritis), 787.91 (diarrhea), 008.6–008.8 (viral gastroenteritis), 001.0–005.9 (cholera, typhoid/paratyphoid fevers, other salmonella infections, shigellosis, and bacterial food poisioning; excluding 003.2 [localized salmonella infections]), 008.0–008.5 (bacterial gastroenteritis), and 006.0–007.9 (amebiasis and other protozoal intestinal disease; excluding 006.3–006.6 [parasitic gastroenteritis]).

A claim identified as the primary discharge diagnosis or 1 of 15 other possible discharge diagnoses from inpatient admission data was classified as a hospitalization. A claim identified in 1 of the 2 diagnosis fields in the outpatient services data was classified as an outpatient visit. Encounters were classified as ER visits (i.e., not hospitalizations or outpatient visits) if “urgent care facility” or “emergency room” was specified in either the inpatient or outpatient services data. Patients evaluated in more than one setting for the same RV episode may have resulted in multiple encounters appearing in the database for the one RV episode.

#### Economic Outcomes

Total, inpatient, outpatient and ER mean costs were calculated for first RV and first diarrhea episodes that occurred within each cohort. For each individual with a first RV episode, the costs incurred during the episode were summed. RV-coded claims dated within 14 days of each other were used to identify the episode of first RV infection. The first RV episode was assumed to last from 14 days before the first RV-coded claim to 14 days after the last RV-coded claim of the episode (unless truncated by end of continuous eligibility). Individuals with no first RV episode had a cost of zero for cost of first RV episode. Similar methods were used to calculate the cost for diarrhea episode.

### Statistical Analysis

#### Overall Cohort Description

For all eligible children, gender, year of eligibility start, health plan type, and race (for Truven Medicaid) were described, as well as vaccination cohort. Frequencies and proportions were reported for categorical variables, while medians and ranges were reported for continuous variables.

#### Clinical Outcomes

Using data from the follow-up of each child, the incidence rates of first RV episodes, RV-coded healthcare encounters and diarrhea-coded healthcare encounters were calculated by dividing the total number of each event by the total person-years of observation for each vaccination cohort. The resulting incidence rates were reported as events per 10,000 persons per year. This person-time approach was used to account for different lengths of observation among study subjects in a non-experimental setting. Poisson regressions were used to generate the incidence rates’ 95% confidence intervals (CIs). Incidence rates were compared between vaccination cohorts by incidence rate ratios.

#### Economic Outcomes

Mean total, inpatient, outpatient and ER costs were calculated for first RV and diarrheal episode by summing first episode costs for a cohort and dividing this by the number of individuals in the cohort. As individuals with no first RV episode had a cost of zero for cost of first RV episode, these individuals contributed a cost of zero to the sum of first episode costs. Since cost data are often not normally distributed, the bootstrapping method was used to estimate the 95% CI of the difference in costs between vaccination cohorts. Bootstrapping methods make no assumptions about data distributions and are thus a valid approach for making statistical inferences [18]. With the bootstrapping method, the patients within the vaccination cohorts were sampled with replacement 500 times. Within each of the 500 resamples, differences in cost between cohorts for RV (and diarrhea) episodes were calculated. The cost differences between vaccination cohorts from the resamples were then used to determine the 95% CIs for the cost differences.

#### Sensitivity Analysis

In a separate analysis we compared outcomes from 6 weeks to 8 months of age between historically unvaccinated children, contemporarily unvaccinated children and children with some RV vaccination prior to 8 months during the vaccination window. This was conducted as a sensitivity analysis as children during this time could be partially vaccinated and not yet eligible, simply due to age, to meet full vaccination according to ACIP guidelines. Each eligible child was observed from his/her estimated birth date to 8 months and 0 days old. Individuals’ person-time in the period from 6 weeks through 8 months old was examined to assess exposure to RV vaccines during the vaccination window, and to assess clinical and economic outcomes during this time. Children who received any RV vaccinations during the vaccination window contributed person-time to the any vaccination cohort. The historically unvaccinated cohort was for individuals who were 8 months old prior to 2007 (and thus reached the end of the vaccination window either in the year that RV vaccine was introduced or else prior) and received no vaccination during the window. The contemporarily unvaccinated cohort was for individuals 8 months old after 2006 with no vaccination during the window. For the period from 6 weeks to 8 months old, claims with the ICD-9 codes for diarrhea and/or RV were identified and used to describe the clinical and economic burden of RV by vaccination status as described for the main analysis.

For all cost analyses, costs were inflation-adjusted to 2012 US dollars based on the medical care component of the Consumer Price Index.

All analyses were performed using SAS software version 9.2 (SAS Institute, Inc., Cary, NC, USA).

## Results

A total of 1,069,485 patients observed for 1,525,125 person-years were included in the analysis of commercial data, and 515,577 patients observed for 837,427 person-years were included in the analysis of Medicaid data (Table 1). Among the commercially insured children, 68.5% were eligible from birth on or after 2007. In the commercial population of children who were eligible for vaccination in 2007 or later, 66.2% of children had received at least one RV vaccine by 8 months of age. Forty four percent had completed the vaccination series per ACIP recommendation, and 22.4% had incomplete vaccination series per ACIP schedule.

**Table 1:** Characteristics of eligible children in Commercial and Medicaid populations.

	Commercial	Medicaid
Characteristic	N = 1,069,485	N = 515,577
Female, n (%)	519,984 (48.6%)	251,101 (48.7%)
Year of continuous eligibility start, n (%)		
2000	4,134 (0.4%)	-
2001	13,357 (1.2%)	-
2002	23,851 (2.2%)	27,406 (5.3%)
2003	46,191 (4.3%)	85,465 (16.6%)
2004	68,294 (6.4%)	81,195 (15.7%)
2005	86,217 (8.1%)	80,399 (15.6%)
2006	95,207 (8.9%)	78,660 (15.3%)
2007	119,314 (11.2%)	38,713 (7.5%)
2008	153,562 (14.4%)	38,177 (7.4%)
2009	204,964 (19.2%)	43,582 (8.5%)
2010	176,773 (16.5%)	41,980 (8.1%)
2011	77,621 (7.3%)	-
Type of health plan, n (%)		
Comprehensive	19,028 (1.8%)	505,581 (98.1%)
Preferred provider organization	810,285 (75.8%)	806 (0.2%)
Other	240,172 (22.5%)	9,190 (1.8%)
Race, n (%)		
White	NR	315,663 (61.2%)
Black	NR	102,454 (19.9%)
Hispanic	NR	70,304 (13.6%)
Other	NR	27,156 (5.3%)
Vaccination cohort distribution, n (%)		
Completed Vaccination	321,615 (30.1%)	21,129 (4.1%)
Incomplete Vaccination	164,733 (15.4%)	31,951 (6.2%)
Historical Unvaccinated	335,090 (31.3%)	351,528 (68.2%)
Contemporary Unvaccinated	248,047 (23.2%)	110,969 (21.5%)

Abbreviations: NR, not reported.

In the Medicaid population, 31.5% were eligible from birth on or after 2007. Thirty two percent of children in this Medicaid population who were eligible for vaccination in 2007 or later had received at least one RV vaccine by 8 months of age. Among those eligible for vaccination during that time, 12.9% had completed the vaccination series per ACIP, and 19.5% had incomplete vaccination series at 8 months of age.

### Clinical Outcomes

As shown in Table 2, among children after 8 months of age in the Commercial population, the incidences of first RV episode per 10,000 persons per year in the complete vaccination cohort (3.3 [95% CI 2.8–3.9]) and incomplete vaccination cohort (4.0 [95% CI 3.3–5.0]) were statistically significantly less than the incidence in the contemporarily unvaccinated cohort (20.9 [95% CI 19.5–22.4]) [Incidence Rate Ratio[IRR] = 0.16 (95% CI 0.13–0.19) and IRR = 0.19 (95% CI 0.16–0.24), respectively]. The incidence per 10,000 persons per year in the contemporarily unvaccinated cohort (20.9 [95% CI 19.5–22.4]) was lower than that in the historically unvaccinated cohort (40.3 [95% CI 38.6–42.1]) [IRR = 0.52 (95% CI 0.48–0.56)].

**Table 2:** Incidence of first RV episode in Commercial and Medicaid populations.

	Commercial			Medicaid		
	Incidence per 10,000 persons per year (95% CI)		Incidence rate ratio (95% CI)	Incidence per 10,000 persons per year (95% CI)		Incidence rate ratio (95% CI)
	[A]	[B]	[A]/[B]	[C]	[D]	[C]/[D]
*Cohort Comparison*						
Contemporary vs. Historical Unvaccinated	Contemporary Unvaccinated	Historical Unvaccinated		Contemporary Unvaccinated	Historical Unvaccinated	
	20.9 (19.5–22.4)	40.3 (38.6–42.1)	0.52 (0.48–0.56)	14.6 (12.8–16.7)	52.0 (50.2–53.8)	0.28 (0.24–0.32)
Completed vs. Contemporary Unvaccinated	Completed Vaccinated	Contemporary Unvaccinated		Completed Vaccinated	Contemporary Unvaccinated	
	3.3 (2.8–3.9)	20.9 (19.5–22.4)	0.16 (0.13–0.19)	7.5 (4.8–11.8)	14.6 (12.8–16.7)	0.52 (0.32–0.83)
Incomplete vs. Contemporary Unvaccinated	Incomplete Vaccinated	Contemporary Unvaccinated		Incomplete Vaccinated	Contemporary Unvaccinated	
	4.0 (3.3–5.0)	20.9 (19.5–22.4)	0.19 (0.16–0.24)	9.0 (6.5–12.3)	14.6 (12.8–16.7)	0.61 (0.44–0.87)

Abbreviations: CI, confidence interval; vs, versus.

Among children after 8 months of age in the Medicaid population, the incidences per 10,000 persons per year in the complete vaccination cohort (7.5 [95% CI 4.8–11.8]) and incomplete vaccination cohort (9.0 [95% CI 6.5–12.3]) were statistically significantly less than the incidence in the contemporarily unvaccinated cohort (14.6 [95% CI 12.8–16.7]) [IRR = 0.52 (95% CI 0.32–0.83) and IRR = 0.61 (95% CI 0.44–0.87), respectively]. The incidence in the contemporarily unvaccinated cohort (14.6 [95% CI 12.8–16.7]) was lower than that in the historically unvaccinated cohort (52.0 [95% CI 50.2–53.8] per 10,000 persons per year) [IRR = 0.28 (95% CI 0.24–0.32)].

As shown in Table 3, among children after 8 months of age in the Commercial population, the RV-related inpatient, outpatient and ER visit rates were significantly lower in the contemporarily unvaccinated cohort compared with the historically unvaccinated cohort as demonstrated by the IRRs less than one. In this age group, those completely vaccinated and incompletely vaccinated both had significantly lower RV-related inpatient, outpatient and ER visit rates compared with the contemporarily unvaccinated cohort. There were similar findings in the Medicaid population for children after 8 months. Except for the comparison of inpatient visits among complete vaccination versus contemporarily unvaccinated, all other IRRs between vaccination cohorts and unvaccinated cohorts were statistically significant.

**Table 3:** Incidence of RV-coded hospitalizations, outpatient visits and ER visits in Commercial and Medicaid populations.

	Commercial			Medicaid		
	Incidence per 10,000 persons per year (95% CI)		Incidence rate ratio (95% CI)	Incidence per 10,000 persons per year (95% CI)		Incidence rate ratio (95% CI)
	[A]	[B]	[A]/[B]	[C]	[D]	[C]/[D]
*Cohort Comparison*						
Contemporary vs. Historical Unvaccinated	Contemporary Unvaccinated	Historical Unvaccinated		Contemporary Unvaccinated	Historical Unvaccinated	
Inpatient visits	12.2 (11.1–13.3)	24.4 (23.1–25.8)	0.50 (0.45–0.56)	7.2 (5.9–8.7)	25.7 (24.5–27.0)	0.28 (0.23–0.34)
Outpatient visits	13.6 (12.5–14.8)	21.6 (20.3–22.9)	0.63 (0.57–0.70)	9.4 (8.0–11.1)	27.2 (25.9–28.5)	0.35 (0.29–0.42)
ER visits	11.2 (10.2–12.3)	17.7 (16.5–18.9)	0.64 (0.57–0.71)	8.0 (6.6–9.6)	42.0 (40.4–43.7)	0.19 (0.16–0.23)
Completed vs. Contemporary Unvaccinated	Completed Vaccinated	Contemporary Unvaccinated		Completed Vaccinated	Contemporary Unvaccinated	
Inpatient visits	1.1 (0.9–1.5)	12.2 (11.1–13.3)	0.09 (0.07–0.13)	5.2 (3.0–8.9)	7.2 (5.9–8.7)	0.72 (0.40–1.28)
Outpatient visits	2.7 (2.3–3.3)	13.6 (12.5–14.8)	0.20 (0.17–0.25)	3.6 (1.9–6.9)	9.4 (8.0–11.1)	0.38 (0.19–0.75)
ER visits	1.1 (0.8–1.5)	11.2 (10.2–12.3)	0.10 (0.07–0.14)	1.6 (0.6–4.2)	8.0 (6.6–9.6)	0.20 (0.07–0.54)
Incomplete vs. Contemporary Unvaccinated	Incomplete Vaccinated	Contemporary Unvaccinated		Incomplete Vaccinated	Contemporary Unvaccinated	
Inpatient visits	1.8 (1.3–2.4)	12.2 (11.1–13.3)	0.15 (0.11–0.20)	4.0 (2.5–6.5)	7.2 (5.9–8.7)	0.56 (0.33–0.93)
Outpatient visits	2.9 (2.3–3.7)	13.6 (12.5–14.8)	0.22 (0.17–0.28)	6.1 (4.2–9.0)	9.4 (8.0–11.1)	0.65 (0.43–0.99)
ER visits	1.4 (1.0–2.0)	11.2 (10.2–12.3)	0.12 (0.09–0.18)	4.3 (2.7–6.7)	8.0 (6.6–9.6)	0.53 (0.33–0.88)

Abbreviations: CI, confidence interval; vs, versus.

The incidence of diarrhea-related resource utilization for both the Commercial and Medicaid populations is described in Table 4. Among children after the age of 8 months in the Commercial population, the diarrhea-related inpatient and outpatient visit rates were significantly lower in the contemporarily unvaccinated cohort compared with the historically unvaccinated cohort as demonstrated by the IRRs less than one. The diarrhea-related ER visit rate was significantly higher in the contemporarily unvaccinated cohort compared with the historically unvaccinated cohort. Those completely vaccinated and incompletely vaccinated both had lower diarrhea-related inpatient and ER visit rates compared with the contemporarily unvaccinated cohort; the opposite was found for the outpatient visit rate. Among children after the age of 8 months in the Medicaid population, the diarrhea-related inpatient, outpatient and ER visit rates were significantly lower in the contemporarily unvaccinated cohort compared with the historically unvaccinated cohort. Diarrhea-related resource utilization was higher for those completely vaccinated and incompletely vaccinated compared with the contemporary unvaccinated.

**Table 4:** Incidence of diarrhea-coded hospitalizations, outpatient visits and ER visits in Commercial and Medicaid populations.

	Commercial			Medicaid		
	Incidence per 10,000 persons per year (95% CI)		Incidence rate ratio (95% CI)	Incidence per 10,000 persons per year (95% CI)		Incidence rate ratio (95% CI)
	[A]	[B]	[A]/[B]	[C]	[D]	[C]/[D]
*Cohort Comparison*						
Contemporary vs. Historical Unvaccinated	Contemporary Unvaccinated	Historical Unvaccinated		Contemporary Unvaccinated	Historical Unvaccinated	
Inpatient visits	53.5(51.2–55.8)	72.7(70.4–75.1)	0.74 (0.70–0.78)	60.4(56.5–64.6)	86.9(84.6–89.2)	0.70 (0.65–0.75)
Outpatient visits	1,845.4(1,832.0–1,858.9)	1,961.5 (1,949.4–1,973.8)	0.94 (0.93–0.95)	1,875.0 (1,852.8–1,897.5)	1,959.2 (1,948.2–1,970.2)	0.96 (0.95–0.98)
ER visits	271.7(266.6–276.9)	253.8(249.5–258.2)	1.07 (1.05–1.10)	547.8(535.9–560.1)	721.3(714.6–728.0)	0.76 (0.75–0.78)
Completed vs. Contemporary Unvaccinated	Completed Vaccinated	Contemporary Unvaccinated		Completed Vaccinated	Contemporary Unvaccinated	
Inpatient visits	29.4(27.7–31.1)	53.5(51.2–55.8)	0.55 (0.51–0.59)	75.0(65.1–86.5)	60.4(56.5–64.6)	1.24 (1.06–1.46)
Outpatient visits	2,068.4(2,054.3–2,082.5)	1,845.4(1,832.0–1,858.9)	1.12 (1.11–1.14)	2,831.6 (2,766.6–2,898.1)	1,875.0 (1,852.8–1,897.5)	1.51 (1.47–1.55)
ER visits	209.0 (204.5–213.5)	271.7 (266.6–276.9)	0.77 (0.75–0.79)	716.3 (684.0–750.2)	547.8 (535.9–560.1)	1.31 (1.24–1.38)
Incomplete vs. Contemporary Unvaccinated	Incomplete Vaccinated	Contemporary Unvaccinated		Incomplete Vaccinated	Contemporary Unvaccinated	
Inpatient visits	37.0(34.5–39.6)	53.5(51.2–55.8)	0.69 (0.64–0.75)	73.0(65.3–81.6)	60.4(56.5–64.6)	1.21 (1.06–1.38)
Outpatient visits	1,918.0(1,899.9–1,936.2)	1,845.4 (1,832.0–1,858.9)	1.04 (1.03–1.05)	2,724.3 (2,675.0–2,774.4)	1,875.0 (1,852.8–1,897.5)	1.45 (1.42–1.49)
ER visits	225.1(219.0–231.4)	271.7(266.6–276.9)	0.83 (0.80–0.86)	708.3(683.4–734.2)	547.8(535.9–560.1)	1.29 (1.24–1.35)

Abbreviations: CI, confidence interval; vs, versus.

### Economic Outcomes

The mean costs per patient of first RV episode for the Commercial and Medicaid populations are shown in Table 5. In the Commercial population among children after the age of 8 months, the mean total cost, inpatient cost, outpatient cost and ER cost was lower for the completely vaccinated versus the contemporary unvaccinated cohort, for the incompletely vaccinated versus the contemporarily unvaccinated cohort, and for the contemporarily unvaccinated versus historically unvaccinated cohort; the differences were statistically significant. The mean cost differences between those who completed vaccination compared to those contemporarily unvaccinated were $15.33(95% CI $12.99-$18.03) and $4.26 (95% CI $2.34-$6.35) per patient in Commercial and Medicaid populations, respectively. Correspondingly, the reduction in cost between those with incomplete vaccination compared to the contemporarily unvaccinated was $14.96 (95% CI $12.63-$17.63) and $3.77 (95% CI $1.34-$5.98) in Commercial and Medicaid populations, respectively. The largest difference in the mean cost of first RV episode was among the inpatient costs among all comparisons.

**Table 5:** Mean cost of first RV episode in Commercial and Medicaid populations.

	Commercial			Medicaid		
	Cost per 1,000 persons Mean ($2012)		Difference ($2012) (95% CI)	Cost per 1,000 persons Mean ($2012)		Difference ($2012)(95% CI)
	[A]	[B]	[A]-[B]	[C]	[D]	[C]-[D]
*Cohort Comparison*						
Contemporary vs. Historical Unvaccinated	Contemporary Unvaccinated	Historical Unvaccinated		Contemporary Unvaccinated	Historical Unvaccinated	
Total costs	17,092	34,659	-17,567 (-20,824; -14,140)	6,025	21,872	-15,846 (-19,339; -12,917)
Inpatient costs	11,698	23,416	-11,717 (-14,296; -8,919)	4,445	14,967	-10,522 (-13,727; -7,832)
Outpatient costs	3,012	6,461	-3,448 (-4,302; -2,644)	1,062	3,821	-2,759 (-3,302; -2,232)
ER costs	2,381	4,783	-2,402 (-2,945; -1,895)	518	3,083	-2,565 (-2,828; -2,294)
Completed vs. Contemporary Unvaccinated	Completed Vaccinated	Contemporary Unvaccinated		Completed Vaccinated	Contemporary Unvaccinated	
Total costs	1,764	17,092	-15,328 (-18,026; -12,992)	1,762	6,025	-4,263 (-6,354; -2,338)
Inpatient costs	1,228	11,698	-10,470 (-12,794; -8,485)	1,365	4,445	-3,079 (-4,846; -1,425)
Outpatient costs	331	3,012	-2,682 (-3,257; -2,213)	275	1,062	-788 (-1,288; -379)
ER costs	205	2,381	-2,176 (-2,492; -1,868)	122	518	-396 (-589; -219)
Incomplete vs. Contemporary Unvaccinated	Incomplete Vaccinated	Contemporary Unvaccinated		Incomplete Vaccinated	Contemporary Unvaccinated	
Total costs	2,128	17,092	-14,964 (-17,629; -12,633)	2,252	6,025	-3,773 (-5,980; -1,342)
Inpatient costs	1,371	11,698	-10,327 (-12,572; -8,415)	1,668	4,445	-2,777 (-4,730; -632)
Outpatient costs	419	3,012	-2,593 (-3,160; -2,071)	406	1,062	-656 (-1,187; -226)
ER costs	338	2,381	-2,044 (-2,408; -1,691)	178	518	-340 (-526; -157)

Abbreviations: CI, confidence interval; vs, versus.

The cost per patient of first diarrhea episode for the Commercial and Medicaid populations is shown in Table 6. In the Commercial population among children after the age of 8 months, the mean total cost, inpatient cost, outpatient cost and ER cost was lower for the contemporarily unvaccinated versus historically unvaccinated cohort, completely vaccinated versus contemporarily unvaccinated cohort, and incompletely vaccinated versus contemporarily unvaccinated cohort; the differences were statistically significant. The mean total cost difference between those who completed vaccination compared to those contemporarily unvaccinated was $34.84 (95% CI $17.69-$49.62) and $21.69 (95% CI $6.57-$37.40) per patient in Commercial and Medicaid populations, respectively. The largest difference in the mean cost of first RV episode was among the inpatient costs for comparisons between completely vaccinated and contemporarily unvaccinated.

**Table 6:** Mean cost of first diarrhea episode in Commercial and Medicaid populations.

	Commercial			Medicaid		
	Cost per 1,000 persons Mean ($2012)		Difference ($2012) (95% CI)	Cost per 1,000 persons Mean ($2012)		Difference ($2012) (95% CI)
	[A]	[B]	[A]-[B]	[C]	[D]	[C]-[D]
*Cohort Comparison*						
Contemporary vs. Historical Unvaccinated	Contemporary Unvaccinated	Historical Unvaccinated		Contemporary Unvaccinated	Historical Unvaccinated	
Total costs	142,194	199,950	-57,756 (-74,267; -41,897)	87,689	130,878	-43,190 (-58,861; -28,705)
Inpatient costs	58,382	82,342	-23,959 (-39,483; -9,416)	43,185	52,497	-9,312 (-24,295; 4,480)
Outpatient costs	57,759	82,610	-24,851 (-27,647; -21,989)	31,540	49,900	-18,360 (-20,286; -16,573)
ER costs	26,052	34,998	-8,946 (-10,345; -7,567)	12,964	28,481	-15,517 (-16,475; -14,333)
Completed vs. Contemporary Unvaccinated	Completed Vaccinated	Contemporary Unvaccinated		Completed Vaccinated	ContemporaryUnvaccinated	
Total costs	107,352	142,194	-34,841 (-49,615; -17,687)	65,998	87,689	-21,691 (-37,397; -6,570)
Inpatient costs	38,716	58,382	-19,666 (-34,484; -3,475)	21,631	43,185	-21,554 (-37,338; -7,763)
Outpatient costs	51,536	57,759	-6,223 (-8,920; -3,788)	30,359	31,540	-1,181 (-3,856; 1,471)
ER costs	17,100	26,052	-8,952 (-10,061; -7,824)	14,008	12,964	1,044 (-486; 2,610)
Incomplete vs. Contemporary Unvaccinated	Incomplete Vaccinated	Contemporary Unvaccinated		Incomplete Vaccinated	Contemporary Unvaccinated	
Total costs	117,875	142,194	-24,319 (-39,921; -7,198)	92,545	87,689	4,856 (-17,142; 32,031)
Inpatient costs	45,770	58,382	-12,612 (-27,612; 3,772)	39,707	43,185	-3,478 (-24,764; 23,022)
Outpatient costs	51,449	57,759	-6,310 (-9,189; -3,504)	38,774	31,540	7,234 (4,184; 10,362)
ER costs	20,656	26,052	-5,396 (-7,112; -3,609)	14,064	12,964	1,100 (-389; 2,465)

Abbreviations: CI, confidence interval; vs, versus.

### Sensitivity Analysis

For this analysis, a total of 1,425,387 patients observed for 674,290 person-years were included in the analysis of Commercial data, and 640,904 patients observed for 311,156 person-years were included in the analysis of Medicaid data.

As shown in S1 Table, among children 6 weeks to 8 months of age in the Commercial population, the incidence of first RV episode per 10,000 persons per year in the any vaccination cohort (5.2 [95% CI 4.5–6.1]) was statistically significantly less than the incidence in the contemporarily unvaccinated cohort (13.8 [95% CI 11.9–15.9]) [IRR = 0.38 (95% CI 0.31–0.47)]. The incidence per 10,000 persons per year in the contemporarily unvaccinated cohort (13.8 [95% CI 11.9–15.9]) was statistically lower than that in the historically unvaccinated cohort (33.0 [95% CI 30.7–35.5]) [IRR = 0.42 (95% CI 0.36–0.49)].

Among children 6 weeks to 8 months of age in the Medicaid population, the incidence of first RV episode per 10,000 persons per year in the any vaccination cohort (18.1 [95% CI 14.2–23.1]) was not statistically significantly different than the incidence in the contemporarily unvaccinated cohort (13.8 [95% CI 11.1–17.2]) [IRR = 1.31 (95% CI 0.95–1.82)]. The incidence in the contemporarily unvaccinated cohort (13.8 [95% CI 11.1–17.2]) was significantly lower than that in the historically unvaccinated cohort (62.3 [95% CI 59.0–65.7] per 10,000 persons per year) [IRR = 0.22 (95% CI 0.18–0.28)].

As shown in S2 Table, among children 6 weeks to 8 months of age in the Commercial population, the RV-related inpatient, outpatient and ER visit rates were significantly lower in the any vaccination cohort compared with the contemporarily unvaccinated and historically unvaccinated cohorts, as demonstrated by the IRRs less than one. In the Medicaid population, the visit rates were not always lower in the any vaccination cohort compared with the contemporarily unvaccinated cohort and were statistically higher for the outpatient visit rate. The visit rates were significantly lower in the any vaccination cohort compared with the historically unvaccinated cohort. The diarrhea-related utilization rates for both populations showed higher utilization in the any vaccination cohort compared with the contemporarily unvaccinated cohort for some types of utilization as well (S3 Table).

The mean total cost, inpatient cost, outpatient cost and ER cost per patient of first RV episode was lower for the any vaccination cohort versus contemporary unvaccinated cohort, and for the any vaccinated cohort versus historically unvaccinated cohort in both the Commercial and Medicaid populations, although statistical significance was not always reached in the comparisons in the Medicaid population (S4 Table). In the Commercial population, the mean total cost, inpatient cost and ER cost per patient of first diarrhea episode was significantly lower for the any vaccination cohort versus contemporary unvaccinated cohort; the mean outpatient cost was significantly higher though. In the Medicaid population, the mean outpatient and ER costs were also significantly higher in the any vaccination cohort versus contemporary unvaccinated cohort. In both populations, the mean costs were significantly lower for the any vaccination cohort versus historical unvaccinated cohort (S5 Table).

## Discussion

In this study, since the introduction of the RV vaccine, only 43.8% of children in the Commercial population and 12.9% of children in the Medicaid population had completed vaccination according to ACIP guidelines (vaccination by 8 months of age). The CDC reported that 43%, 59% and 67.3% of children aged 19 to 35 months were fully vaccinated against rotavirus in 2009, 2010 and 2011 respectively [9,19]. CDC also reported that in 2009, 39% of infants had been vaccinated with RV vaccine by 7 months of age [19]. It is important to note that data for the vaccination cohorts in the current study covers 2007–2011 for the Commercial population and 2007–2010 for the Medicaid population, thus trends in vaccination during these years make it difficult to compare vaccination coverage to that reported in any particular year by CDC. Also, complete vaccination coverage reported by CDC required that the correct number of doses be given to the child, while vaccination coverage in the current study required that the correct number of doses be given to the child within the ACIP recommended vaccination window.

Findings from the current study show that RV vaccination within the ACIP recommended vaccination window, including those achieved by mixed vaccination, results in significant reduction in RV infection for children after 8 months old. The analysis of the Commercial population shows that the incidence of RV infection among contemporarily unvaccinated children was more than five times higher than among completely vaccinated children. In the Medicaid analysis, RV infection incidence among contemporarily unvaccinated children was two-fold higher than among completely vaccinated children. Similar trends were seen with RV-related healthcare resource utilization. The findings in this study on decline of RV infection following vaccination are in agreement with other published studies [10–13].

This current study also reports that the incidence of RV infection among contemporarily unvaccinated children was higher than among incompletely vaccinated children after 8 months old. In a claims analysis, Wang et al. [12] observed that incomplete *Rotateq*^®^ vaccination exhibits effectiveness against rotavirus. However, it must be noted that both these studies are observational. The current study was not designed to evaluate incomplete vaccination. Incomplete vaccination is not recommended by ACIP as it has not been evaluated in clinical trials. Furthermore, some children categorized as incompletely vaccinated in the current study went on to complete vaccination outside the ACIP recommended vaccination window; in the Commercial population 7,017 (4%) and in the Medicaid population 7,160 (22%) of those incompletely vaccinated did complete vaccination off schedule. These children may have had benefits more similar to completely vaccinated children, albeit delayed. It is also possible that some of the benefit observed in the incompletely vaccinated group in the current study is due to herd immunity gained from others being completely vaccinated.

Comparisons of outcomes between the contemporary cohorts- that is, completely vaccinated, incompletely vaccinated and contemporarily unvaccinated- should be made while considering that inherent differences between groups may contribute to differences in outcomes between these groups. Healthcare seeking behavior in general may be strongest among the completely vaccinated and weakest among the contemporarily unvaccinated, with the incompletely vaccinated somewhere in between those two groups. Individuals who seek vaccination may be more likely to seek wellness visits and sick visits. Thus, completely vaccinated individuals may have seemingly similar rates of RV infection as those incompletely vaccinated, but this could be due to completely vaccinated individuals being more likely to seek care when they are not well and thus have a greater likelihood of having a claim for RV infection.

Another important consideration when comparing outcomes across cohorts is the relatively short follow-up time. Among the historically unvaccinated cohort, average follow-up was 1.5 years and 1.8 years in the Commercial and Medicaid populations, respectively. Among contemporary vaccination cohorts, average follow-up ranged from 1.2 to 1.6 years in the Commercial population and 1.2 to 1.3 years in the Medicaid population. In the Commercial cohort, 11% of children born in the post-RV vaccination era were born in 2011 which was the last year of data availability. Likewise, in Medicaid, one quarter of children were born in 2010, the last year of data availability for that database. Firstly, a short follow-up period in the contemporary cohorts limits the ability to detect waning immunity. With this relatively low average length of follow-up, waning of immunity conferred by vaccination occurring several years after vaccination would not have been detected in the current study. Thus, the benefits of complete and incomplete vaccination reported here may not apply to immunity five years following vaccination. Secondly, a reduction in infection rate of any pathogen, as commonly seen with widespread vaccination, is associated with an increased average age in the population of acquiring that particular infection [20]. With the introduction of RV vaccination, the average age of RV infection may have increased. The relatively short average length of follow-up in this study may not have been long enough to reveal later infections occurring in the vaccinated cohorts.

In both the Commercial and Medicaid populations, the rate of RV was at least two fold higher among historically than contemporarily unvaccinated children for children after 8 months of age, indicating a possible strong indirect effect of the vaccine. Children in the historically and contemporarily unvaccinated cohorts were similar in terms of regional distribution and insurance type (for the Commercial population) as well as having been observed for the same age period. A prior observational study by Cortes et al. [10] demonstrated an indirect benefit of RV vaccination for 2007–2008 but not for 2008–2009. The current study evaluated indirect effect for the entire post-vaccination period which was longer than that observed in the study by Cortes et al. [10].

Although the study was not designed to compare rates between Medicaid and Commercial populations, this study noted lower reduction of disease for the vaccinated versus unvaccinated in the Medicaid population compared to the Commercial population. The lack of race data in the Commercial database and lack of socio-economic status data (SES) in both databases makes it impossible to adjust for differences and potential confounders. Other studies have noted less benefit for oral RV vaccination with lower SES when comparing results across countries [21]. Clinical trials conducted in various global locations showed that in middle income settings in Latin America, South Africa and Vietnam vaccine efficacy ranged from 72% to 83%; in low income settings in Asia and Africa vaccine efficacy was lower, ranging from 39% to 49% [22–25]. It has been hypothesized that the poorer response in these lower SES settings may be due to such factors as a poor immune response or a very high incidence rate that overwhelms the immunity that vaccination is meant to confer [21]. It is unknown whether such differences exist to the same extent between SES settings in the US and whether these would have contributed to the difference in disease reduction observed in the Medicaid versus Commercial population.

Some of the findings on diarrhea-related resource utilization showed unanticipated results. For example, among children after the age of 8 months in the Commercial population, diarrhea-related outpatient visits were significantly higher among the vaccinated versus contemporarily unvaccinated while the opposite was found for inpatient and ER visits. In Medicaid, inpatient, outpatient and ER visit rates for the vaccinated were higher than the contemporarily unvaccinated. This may have occurred if vaccinated children were more likely than unvaccinated children to be taken for a healthcare visit upon the appearance of any diarrheal symptoms which include infection other than RV. Similar to what was stated previously, vaccinated children may be more likely than unvaccinated children in general to exhibit healthcare seeking behavior. It must be remembered that this outcome was measured by all events with any indication for diarrhea and not specifically RV, so higher diarrhea-related resource utilization among the vaccinated could reflect greater healthcare seeking behavior for these non-RV infections. Thus, this outcome is more difficult to interpret than the outcome with RV specific coding.

The economic analysis of RV burden by vaccination cohort demonstrated lower cost burden as well for both complete and incomplete vaccination relative to unvaccinated children. In the Commercial analysis for children after the age of 8 months, the mean total cost per cohort member for first RV episode was nearly $15 greater for unvaccinated children compared with completely and incompletely vaccinated children. For a birth cohort of 3,952,841 children, the size of the US birth cohort in 2012 [26], this translates to a savings in treating first RV episodes of nearly 60 million dollars when all are vaccinated. The mean total cost per cohort member for first RV episode was approximately $17 greater for unvaccinated children prior to the availability of vaccination compared with unvaccinated children after the availability of vaccine. Savings and similar trends for the same comparisons were seen in the Medicaid population.

The current study focuses on RV-related and diarrhea-related events among vaccination cohorts after 8 months of age as vaccination should have been completed by the age of 8, according to ACIP guidelines. The burden of RV disease among children younger than 8 months of age is also a concern. Before the availability of vaccination, it was estimated that one in five children hospitalized with RV in the US was less than six months of age [27]. In the sensitivity analysis, we did compare outcomes before 8 months of age between historically unvaccinated children, contemporarily unvaccinated children and children with some RV vaccination prior to 8 months. In the analysis of the Commercial population, the rate of RV incidence was significantly higher in the historically unvaccinated cohort versus the contemporarily unvaccinated cohort, and the rate of RV incidence was significantly higher in the contemporarily unvaccinated cohort versus the cohort with any RV vaccination prior to 8 months. In the analysis of the Medicaid population, the trend was similar for the any vaccination versus historically unvaccinated cohort; however, the rate of RV incidence was not higher in the contemporarily unvaccinated cohort versus the cohort with any RV vaccination prior to 8 months. The RV-related and diarrhea-related utilization rates were lower in the any vaccination cohort compared with the historical unvaccinated cohort but sometimes higher in the any vaccination cohort compared with the contemporary unvaccinated cohort prior to 8 months of age. Comparing outcomes between cohorts prior to 8 months of age is difficult though, as individuals may be in the process of receiving vaccination during this time. An individual who receives vaccination during this time in accordance with ACIP guidelines, for example, can have a period of no vaccination followed by partial vaccination followed by complete vaccination once the series is complete. Thus, RV outcomes prior to 8 months for individuals with any RV vaccination during this time include outcomes that may have occurred during a time of no vaccination, partial or full vaccination. The choice for the current study to focus on outcomes occurring after the age of 8 months, the end of the ACIP-recommended vaccination window, is consistent with prior research published by Payne et al. [28].

There are some limitations of the current study to consider. First, we examined data from only three and four post-RV vaccine seasons in Medicaid (2007–2010) and Commercial (2007–2011) populations, respectively, and cannot be certain that observed changes were due solely to vaccine use. Secular trends in the incidence of RV and other diarrheal pathogens could affect our findings. Second, although we conducted stratified analysis by age and considered pre/post-RV vaccine availability in our analysis of unvaccinated children, we may not have accounted for all confounders; as RV spreads easily among infants and young children [29] factors such as having siblings or daycare attendance could influence risk of RV and could have confounded the observed associations. As with all claims database analyses, ICD-9 codes were used to identify diagnoses; these codes may not reflect confirmed clinical diagnoses and lack information to assess severity of illness. Moreover, medical services obtained outside of a patient’s plan are not captured in a claims database. Thus, there is the potential for exposure misclassification if an individual received vaccination outside a health plan. In addition, resource utilization and costs in this study may be underestimated if a patient sought care outside his or her plan. Additionally the inability in both the data sources to adjust for SES must be considered as a limitation.

In conclusion, this study utilizes data from more than two million children in both Commercial and Medicaid settings and adds to our understanding of the benefits of RV vaccination. RV vaccination confers benefits both in terms of reduction in RV episodes and RV-related resource utilization. In addition, average patient costs due to RV and diarrheal episodes are substantially less among RV vaccinated cohorts in both populations. Vaccination also confers benefits to unvaccinated individuals in the population.

## Supporting Information

S1 Table Incidence of first RV episode in Commercial and Medicaid populations, 6 weeks- 8 months of age. (DOCX)

S2 Table Incidence of RV-coded hospitalizations, outpatient visits and ER visits in Commercial and Medicaid populations, 6 weeks- 8 months of age. (DOCX)

S3 Table Incidence of diarrhea-coded hospitalizations, outpatient visits and ER visits in Commercial and Medicaid populations, 6 weeks- 8 months of age. (DOCX)

S4 Table Mean cost of first RV episode in Commercial and Medicaid populations, 6 weeks- 8 months of age. (DOCX)

S5 Table Mean cost of first diarrhea episode in Commercial and Medicaid populations, 6 weeks- 8 months of age. (DOCX)
