# Supplementary material for: Public Health Impact of Complete and Incomplete Rotavirus Vaccination among Commercially and Medicaid Insured Children in the United States
Source: PLoS One. 2016 Jan 11;11(1):e0145977. doi: 10.1371/journal.pone.0145977 (PMC4709043; doi:10.1371/journal.pone.0145977)
Supplement: S1 Table — (DOCX) [file pone.0145977.s001.docx]

S1 Table. Incidence of first RV episode in Commercial and Medicaid populations, 6 weeks- 8 months of age

|  | Commercial | | | Medicaid | | |
| --- | --- | --- | --- | --- | --- | --- |
|  | Incidence per 10,000 persons per year (95% CI) | | Incidence  rate ratio (95% CI) | Incidence per 10,000 persons per year (95% CI) | | Incidence rate ratio (95% CI) |
|  | [A] | [B] | [A]/[B] | [C] | [D] | [C]/[D] |
| *Cohort Comparison* |  |  |  |  |  |  |
| Any Vaccination Before 8 Months vs. Contemporary Unvaccinated | Any Vaccination | Contemporary Unvaccinated |  | Any Vaccination | Contemporary Unvaccinated |  |
|  | 5.2 (4.5-6.1) | 13.8 (11.9-15.9) | 0.38  (0.31-0.47) | 18.1 (14.2-23.1) | 13.8 (11.1-17.2) | 1.31  (0.95-1.82) |
|  |  |  |  |  |  |  |
| Any Vaccination Before 8 Months vs. Historical Unvaccinated | Any Vaccination | Historical Unvaccinated |  | Any Vaccination | Historical Unvaccinated |  |
|  | 5.2 (4.5-6.1) | 33.0 (30.7-35.5) | 0.16  (0.14-0.19) | 18.1 (14.2-23.1) | 62.3 (59.0-65.7) | 0.29  (0.23-0.37) |
|  |  |  |  |  |  |  |
| Abbreviations: CI, confidence interval; vs., versus. | | | | | | |
