# Supplementary material for: Public Health Impact of Complete and Incomplete Rotavirus Vaccination among Commercially and Medicaid Insured Children in the United States
Source: PLoS One. 2016 Jan 11;11(1):e0145977. doi: 10.1371/journal.pone.0145977 (PMC4709043; doi:10.1371/journal.pone.0145977)
Supplement: S2 Table — (DOCX) [file pone.0145977.s002.docx]

**S2 Table. Incidence of RV-coded hospitalizations, outpatient visits and ER visits in Commercial and Medicaid populations, 6 weeks- 8 months of age**

|  | Commercial | | | Medicaid | | |
| --- | --- | --- | --- | --- | --- | --- |
|  | Incidence per 10,000 persons per year (95% CI) | | Incidence  rate ratio  (95% CI) | Incidence per 10,000 persons per year (95% CI) | | Incidence  rate ratio  (95% CI) |
|  | [A] | [B] | [A]/[B] | [C] | [D] | [C]/[D] |
| *Cohort Comparison* |  |  |  |  |  |  |
| Any Vaccination Before 8 Months vs. Contemporary Unvaccinated | Any Vaccination | Contemporary Unvaccinated |  | Any Vaccination | Contemporary Unvaccinated |  |
| Inpatient visits | 1.9  (1.4-2.4) | 7.5  (6.2-9.1) | 0.25 (0.18-0.34) | 11.8  (8.8-15.9) | 7.9  (5.9-10.5) | 1.49  (0.99-2.26) |
| Outpatient visits | 4.9  (4.2-5.8) | 9.4  (7.9-11.2) | 0.52 (0.41-0.66) | 12.1  (9.0-16.2) | 7.6  (5.7-10.2) | 1.59  (1.05-2.42) |
| ER visits | 1.9  (1.5-2.4) | 7.6  (6.3-9.3) | 0.25 (0.18-0.34) | 7.4  (5.1-10.8) | 7.6  (5.7-10.2) | 0.98  (0.61-1.58) |
|  |  |  |  |  |  |  |
| Any Vaccination Before 8 Months vs. Historical Unvaccinated | Any Vaccination | Historical Unvaccinated |  | Any Vaccination | Historical Unvaccinated |  |
| Inpatient visits | 1.9  (1.4-2.4) | 18.1  (16.4-19.9) | 0.10 (0.08-0.14) | 11.8  (8.8-15.9) | 28.8  (26.6-31.1) | 0.41  (0.30-0.56) |
| Outpatient visits | 4.9  (4.2-5.8) | 19.8  (18.1-21.7) | 0.25 (0.21-0.30) | 12.1  (9.0-16.2) | 35.6  (33.1-38.2) | 0.34  (0.25-0.46) |
| ER visits | 1.9  (1.5-2.4) | 13.4  (12.0-15.0) | 0.14 (0.11-0.19) | 7.4  (5.1-10.8) | 50.8  (47.9-53.9) | 0.15  (0.10-0.21) |
|  |  |  |  |  |  |  |
|  |  |  |  |  |  |  |
| Abbreviations: CI, confidence interval; vs., versus. | | | | | | |
