# Supplementary material for: Public Health Impact of Complete and Incomplete Rotavirus Vaccination among Commercially and Medicaid Insured Children in the United States
Source: PLoS One. 2016 Jan 11;11(1):e0145977. doi: 10.1371/journal.pone.0145977 (PMC4709043; doi:10.1371/journal.pone.0145977)
Supplement: S3 Table — (DOCX) [file pone.0145977.s003.docx]

S3 Table. Incidence of diarrhea-coded hospitalizations, outpatient visits and ER visits in Commercial and Medicaid populations, 6 weeks- 8 months of age

|  | Commercial | | | Medicaid | | |
| --- | --- | --- | --- | --- | --- | --- |
|  | Incidence per 10,000 persons per year (95% CI) | | Incidence rate ratio (95% CI) | Incidence per 10,000 persons per year (95% CI) | | Incidence rate ratio (95% CI) |
|  | [A] | [B] | [A]/[B] | [C] | [D] | [C]/[D] |
| *Cohort Comparison* |  |  |  |  |  |  |
| Any Vaccination Before 8 Months vs. Contemporary Unvaccinated | Any Vaccination | Contemporary Unvaccinated |  | Any Vaccination | Contemporary Unvaccinated |  |
| Inpatient visits | 49.8  (47.4-52.3) | 70.1  (65.7-74.7) | 0.71 (0.66-0.77) | 146.7  (134.8-159.7) | 97.9  (90.2-106.2) | 1.50 (1.33-1.69) |
|  |  |  |  |  |  |  |
| Outpatient visits | 2,010.8  (1,995.2-2,026.4) | 1,911.5 (1,888.2-1,935.1) | 1.05 (1.04-1.07) | 2,656.9 (2,604.5-2,710.4) | 1,764.4 (1,730.9-1,798.5) | 1.51 (1.46-1.55) |
|  |  |  |  |  |  |  |
| ER visits | 231.4  (226.2-236.8) | 288.6  (279.7-297.9) | 0.80 (0.77-0.83) | 827.0  (798.0-857.1) | 565.1  (546.3-584.6) | 1.46 (1.39-1.54) |
|  |  |  |  |  |  |  |
| Any Vaccination Before 8 Months vs. Historical Unvaccinated | Any Vaccination | Historical Unvaccinated |  | Any Vaccination | Historical Unvaccinated |  |
| Inpatient visits | 49.8  (47.4-52.3) | 78.8  (75.2-82.5) | 0.63 (0.59-0.68) | 146.7  (134.8-159.7) | 152.8  (147.7-158.1) | 0.96 (0.88-1.05) |
| Outpatient visits  ER visits | 2,010.8  (1,995.2-2,026.4)  231.4  (226.2-236.8) | 2,212.5  (2,193.2-2,232.1)  262.6  (255.9-269.3) | 0.91 (0.90-0.92)  0.88  (0.85-0.91) | 2,656.9 (2,604.5-2,710.4)  827.0  (798.0-857.1) | 2,601.3  (2,579.9-2,623.0)  963.7  (950.7-976.9) | 1.02 (1.00-1.04)  0.86  (0.83-0.89) |
| Abbreviations: CI, confidence interval; vs, versus. | | | | | | |
