# Supplementary material for: Public Health Impact of Complete and Incomplete Rotavirus Vaccination among Commercially and Medicaid Insured Children in the United States
Source: PLoS One. 2016 Jan 11;11(1):e0145977. doi: 10.1371/journal.pone.0145977 (PMC4709043; doi:10.1371/journal.pone.0145977)
Supplement: S4 Table — (DOCX) [file pone.0145977.s004.docx]

S4 Table. Mean cost of first RV episode in Commercial and Medicaid populations, 6 weeks- 8 months of age

|  | | | | | | | | | | | | | | | | | | | | | | | |  |  |
| --- | --- | --- | --- | --- | --- | --- | --- | --- | --- | --- | --- | --- | --- | --- | --- | --- | --- | --- | --- | --- | --- | --- | --- | --- | --- |
|  | Commercial | | | | | | | | Medicaid | | | | | | | | | | | | | | |  |  |
|  | Cost per 1,000 persons  Mean($2012) | | | | Difference($2012)  (95% CI) | | | | Cost per 1,000 persons Mean($2012) | | | | | | | Difference($2012)  (95% CI) | | | | | |  |  |  |  |
|  | [A] | [B] | | | [A]-[B] | | | | [C] | | | [D] | | | | [C]-[D] | | | | | |  |  |  |  |
| *Cohort Comparison* |  | | |  | | |  | |  | | | | |  | | | | | |  | | | | | |
| Any Vaccination Before 8 Months vs. Contemporary Unvaccinated | Any Vaccination | Contemporary Unvaccinated | | |  | | | | Any Vaccination | | | Contemporary Unvaccinated | | | |  | |  |  |  |  |  |  |  |  |
| Total costs | 2,352 | 13,217 | | | -10,865 (-13,445; -8,631) | | | | 5,099 | | | 5,857 | | | | -757 (-3,141; 1,848) | | | | | |  |  |  |  |
| Inpatient costs | 1,597 | 9,350 | | | -7,752 (-10,021; -5,780) | | | | 4,164 | | | 4,563 | | | | -400 (-2,599; 2,119) | | | | | |  |  |  |  |
| Outpatient costs | 459 | 2,110 | | | -1,651 (-2,026; -1,305) | | | | 629 | | | 821 | | | | -191 (-473; 105) | | | | | |  |  |  |  |
| ER costs | 295 | 1,757 | | | -1,462 (-1,761; -1,195) | | | | 306 | | | 473 | | | | -167 (-350; -7) | | | | | |  |  |  |  |
|  |  | |  | | |  | | |  | | | |  | | | | | |  | | | | | |  |
| Any Vaccination Before 8 Months vs. Historical Unvaccinated | Any Vaccination | Historical Unvaccinated | | |  | | | Any Vaccination | | | Historical  Unvaccinated | | | |  | | | | | | | |  |  |  |
| Total costs | 2,352 | 35,895 | | | -33,543 (-35,840; -31,265) | | | 5,099 | | | 28,945 | | | | -23,846 (-28,730;-19,444) | | | | | | | |  |  |  |
| Inpatient costs | 1,597 | 24,928 | | | -23,331 (-25,487; -21,396) | | | 4,164 | | | 21,022 | | | | -16,858 (-21,563; -12,384) | | | | | | | |  |  |  |
| Outpatient costs | 459 | 6,226 | | | -5,767 (-6,281; -5,256) | | | 629 | | | 4,475 | | | | -3,846 (-4,234; -3,475) | | | | | | | |  |  |  |
| ER costs | 295 | 4,740 | | | -4,444 (-4,792; -4,093) | | | 306 | | | 3,448 | | | | -3,142 (-3,366; -2,931) | | | | | | | |  |  |  |
|  |  | |  | | |  | | |  |  | | | | | | |  | | | |  |  |  |  |  |
| Abbreviations: CI, confidence interval ; vs, versus. | | | | | | | | | | | | | | | | | | | | | | | |  |  |
