# Supplementary material for: Public Health Impact of Complete and Incomplete Rotavirus Vaccination among Commercially and Medicaid Insured Children in the United States
Source: PLoS One. 2016 Jan 11;11(1):e0145977. doi: 10.1371/journal.pone.0145977 (PMC4709043; doi:10.1371/journal.pone.0145977)
Supplement: S5 Table — (DOCX) [file pone.0145977.s005.docx]

S5 Table. Mean cost of first diarrhea episode in Commercial and Medicaid populations, 6 weeks- 8 months of age

|  | | | | | | | | | | | | | | | | | | | | | | |  |  |
| --- | --- | --- | --- | --- | --- | --- | --- | --- | --- | --- | --- | --- | --- | --- | --- | --- | --- | --- | --- | --- | --- | --- | --- | --- |
|  | Commercial | | | | | | | Medicaid | | | | | | | | | | | | | | |  |  |
|  | Cost per 1,000 persons  Mean($2012) | | | | Difference($2012)  (95% CI) | | | Cost per 1,000 persons Mean($2012) | | | | | | | Difference($2012)  (95% CI) | | | | | |  |  |  |  |
|  | [A] | [B] | | | [A]-[B] | | | [C] | | | [D] | | | | [C]-[D] | | | | | |  |  |  |  |
| *Cohort Comparison* |  | | |  | | |  |  | | | | |  | | | | | |  | | | | | |
| Any Vaccination Before 8 Months vs. Contemporary Unvaccinated | Any Vaccination | Contemporary Unvaccinated | | |  | | | Any Vaccination | | | Contemporary Unvaccinated | | | |  | |  |  |  |  |  |  |  |  |
| Total costs | 161,285 | 188,985 | | | -27,700 (-44,622; -10,431) | | | 144,543 | | | 161,021 | | | | -16,479 (-57,143; 19,755) | | | | | |  |  |  |  |
| Inpatient costs | 68,184 | 101,955 | | | -33,771 (-49,944; -17,006) | | | 75,029 | | | 104,298 | | | | -29,269 (-69,322; 7,156) | | | | | |  |  |  |  |
| Outpatient costs | 69,194 | 60,654 | | | 8,540 (6,765; 10,240) | | | 49,085 | | | 39,281 | | | | 9,804 (7,027; 12,251) | | | | | |  |  |  |  |
| ER costs | 23,908 | 26,376 | | | -2,468 (-3,772; -1,257) | | | 20,429 | | | 17,442 | | | | 2,986 (1,626; 4,139) | | | | | |  |  |  |  |
|  |  | |  | | |  | |  | | | |  | | | | | |  | | | | | |  |
| Any Vaccination Before 8 Months vs. Historical Unvaccinated | Any Vaccination | Historical Unvaccinated | | |  | | | Any Vaccination | | Historical  Unvaccinated | | | |  | | | | | | | |  |  |  |
| Total costs | 161,285 | 251,253 | | | -89,967 (-103,765; -75,973) | | | 144,543 | | 220,976 | | | | -76,434 (-96,745; -51,277) | | | | | | | |  |  |  |
| Inpatient costs | 68,184 | 111,400 | | | -43,216 (-56,661; -29,627) | | | 75,029 | | 110,236 | | | | -35,206 (-55,009; -10,460) | | | | | | | |  |  |  |
| Outpatient costs | 69,194 | 101,396 | | | -32,202 (-35,007; -29,732) | | | 49,085 | | 71,560 | | | | -22,475 (-24,848; -20,172) | | | | | | | |  |  |  |
| ER costs | 23,908 | 38,456 | | | -14,549 (-15,579; -13,565) | | | 20,429 | | 39,181 | | | | -18,752 (-19,786; -17,842) | | | | | | | |  |  |  |
|  |  | |  | | |  | |  |  | | | | | | |  | | | |  |  |  |  |  |
| Abbreviations: CI, confidence interval ; vs, versus. | | | | | | | | | | | | | | | | | | | | | | |  |  |
